# HIV/AIDS and the health of older people in the slums of Nairobi, Kenya: results from a cross sectional survey

**DOI:** 10.1186/1471-2458-9-153

**Published:** 2009-05-27

**Authors:** Catherine Kyobutungi, Alex C Ezeh, Eliya Zulu, Jane Falkingham

**Affiliations:** 1 African Population & Health Research Center, P.O Box 10787, GPO 00100, Nairobi, Kenya; 2 Centre for Global Health, Population, Poverty and Policy (GHP3), University of Southampton, Southampton, UK

## Abstract

**Background:**

The proportion of older people is increasing worldwide. Globally, it is estimated that older people (those 60 years or older) constitute more than 11% of the population. As the HIV/AIDS pandemic rages in sub-Saharan Africa (SSA), its impact on older people needs closer attention given the increased economic and social roles older people have taken on as a result of increased mortality among adults in the productive age groups. Few studies have looked at older people and their health in SSA or indeed the impact of HIV/AIDS on their health. This study aims to assess the effect of being directly or indirectly affected by HIV/AIDS on the health of older people in two Nairobi slums.

**Methods:**

Data were collected from residents of the Nairobi Urban Health and Demographic Surveillance area aged 50 years and above on 1^st ^October 2006. Health status was assessed using the short SAGE (Study on Global AGEing and Adult Health) form and two outcome measures – self-rated health and a composite health score – were generated. To assess HIV/AIDS affected status, respondents were asked: *Have you personally been affected by HIV/AIDS? *If yes, a follow up question: "*How have you been personally affected by HIV/AIDS?" *was asked. Ordinallogistic regression was used in models with self-rated health and linear regression in models with the health score.

**Results:**

About 18% of respondents reported being affected by HIV/AIDS in at least one way, although less than 1% reported being infected with HIV. Nearly 60% of respondents reported being in good health, 27% in fair health and 14% in poor health. The overall mean health score was 70.6 (SD: 13.9) with females reporting worse health outcomes than males.

Respondents directly or indirectly affected by HIV/AIDS reported worse health outcomes than those not affected: mean health score: 68.5 and 71.1 respectively (t = 3.21, p = 0.0007), and an adjusted odds ratio of reporting poor health of 1.42 (95%CI: 1.12–1.80).

**Conclusion:**

Poor health outcomes among older people affected by HIV/AIDS highlight the need for policies that target them in the fight against HIV/AIDS if they are to play their envisaged care giving and other traditional roles.

## Background

The proportion of older people is increasing worldwide. It is estimated that older people (those 60 years or older) constitute more than 11% of the population globally, over 20% of the population in developed nations and about 8% in developing ones. The proportion of older people in developing countries is expected to rise to about 20% by 2050 [1]. Older people therefore will increasingly form an important sub-group in numeric terms in developing nations.

Older people have traditionally been held in high esteem in many African societies for their wisdom, role as heads of families, and roles in conflict resolution. Some authors have used the term "gerontocracy" to illustrate the powerful position older people hold in most African societies [2] in [3]. More recently, older people have been engaged in the fight against HIV/AIDS especially in their role as caregivers for HIV infected family members and orphans left behind by deceased relatives [4,5]. The vulnerability of older people as a consequence of the HIV/AIDS epidemic has increased due to the weakening of traditional social support structures, increased mortality of family members in the productive age group and subsequent loss of economic support for older people. In many cases the death of an adult in the reproductive age group is soon followed by the death of the spouse leaving behind a number of orphans. It is estimated that up to 60% of orphaned children live in grandparent-headed households in some SSA countries [6,7].

The proportion of HIV-infected older people has increased in recent years in developed countries where use of antiretroviral therapy is widespread [8-11] but there are indications that this is also the case in some African countries [12,13]. In Kenya, it is estimated that 5% of those infected with HIV/AIDS are aged 50 years and older [14] and recent research and public health discourses are increasingly highlighting the need to focus on older people in the fight against HIV/AIDS [9,15,16]. As the HIV/AIDS pandemic rages in sub-Saharan Africa, its impact on older people needs closer attention because the intersection of the HIV/AIDS pandemic and population aging in SSA may have far reaching consequences on societies' economic, social and political spheres of life. Despite this evident need, issues of aging in Africa have only recently started receiving attention in research and in policies. There is a near absence of policies and programs targeting older people in most countries in SSA and most health policies are geared towards the needs of traditionally vulnerable groups of women and children.

The impact of the HIV/AIDS pandemic on other age groups such as young children, adolescents and adults in reproductive age has been extensively studied [7,17]. There are few studies on older people in SSA in general and their health in particular. It is therefore unsurprising that not much is known about the impact of HIV/AIDS on their health. Studies on the impact of HIV/AIDS on older people have focused on quantifying the extent to which older people are involved in care giving roles [4,7,18], the economic impact of the loss of adult family members [19,20], and the impact of HIV/AIDS on traditional social support networks in the African context including disruptions in living arrangements [5,21]. Some studies have also looked at the multidimensional and indirect effects of HIV/AIDS on older people, including the health consequences of care giving roles [20,21] though not in great detail. The few studies that have assessed the impact of HIV/AIDS on the health and wellbeing of older people have predominantly focused on the indirect effect of HIV/AIDS and have found that it has negative consequences on the health of older people [18,20,21]. A study in Zimbabwe found that only 30% of older people affected by HIV/AIDS reported being in good or very good health and of those in bad or very bad health, 58% attributed it to providing care to AIDS-affected family members [20]. In Uganda, a study on older caregivers found that most respondents had anxiety about their future and wellbeing and that most, especially females, had physical ailments [4]. Similar findings were found in Thailand among older people that were caring for adult children with AIDS, although respondents were only asked about pre-determined health conditions [22]. They reported more anxiety, more insomnia, less happiness than those who were not looking after adult children with HIV/AIDS. They also reported relatively high fatigue, muscle strain, headache and stomachaches. These studies, however, had methodological limitations that preclude their generalizability. These limitations include purposive sampling of only HIV/AIDS affected older people [20] and use of only qualitative methods [4].

This study therefore aims to fill existing knowledge gaps on the impact of HIV/AIDS on the health of older people. It assesses the direct and indirect effects of HIV/AIDS on the health of older people in two Nairobi slums in a quantitative manner using standard measures of health and considering other dimensions in which older people can be affected by HIV/AIDS.

## Methods

Data were collected from all older people residing in the two slum communities where the African Population and Health Research Center (APHRC) is implementing the longitudinal Nairobi Urban Health and Demographic Surveillance System (NUHDSS). The NUHDSS covers the two slums of Korogocho and Viwandani in Nairobi, Kenya's capital and commercial centre. The two slums are located about 5–10 km from the city centre and occupy an area of 0.45 and 0.52 km^2 ^respectively. The informal, and hence non-permanent, nature of the slums settlements encourages the official neglect of these communities in the provision of infrastructure and social services. There are only three public health facilities serving the two communities, but these are located on the outskirts of the slums, serve big catchment areas, are closed at night, and often lack drugs and supplies. About three quarters of the residents buy water from vendors and only 22% have access to piped water compared to 78% in the rest of Nairobi. Only 7% of residents have a flush toilet, 84% use pit latrines and the rest have no toilet facility [23] HIV/AIDS related morbidity and mortality are high; HIV prevalence is estimated at 11.5% [24], and close to 50% of the population aged 5 years and above are dying from AIDS and Tuberculosis combined [25].

The NUHDSS has been operational since January 2003 and data on core demographic events (birth, death, in-migration and out-migration) are collected from members of about 22,000 households in the defined geographic areas, and updated every four months during routine Demographic Surveillance System (DSS) rounds. A total of 2696 older people (those who were aged 50 years and above as of 1^st ^October 2006) were identified as ever being resident in the surveillance areas from the most up-to-date NUHDSS database. Out of these, complete information was collected from 2078 respondents, who were found in their homes after repeated visits and who consented to participate in the study; 102 people refused to participate.

The age of 50 years is thought to incorporate the chronological, functional and social definitions of "old" in Africa and has been adapted by the WHO for its Minimum Data Set project [26]. This age cut-off has also been used in other studies in Africa [20]. Data were collected between November 2006 and February 2007 in the framework of a larger study on the linkages between urbanization, migration, poverty and health outcomes over the life course. An interviewer-administered questionnaire was used to collect data on socioeconomic and demographic characteristics, living arrangements, history of caring for persons with chronic illnesses including HIV/AIDS and caring for children below 15 years of age and health status. Ethical approval was obtained from the Kenya Medical Research Institute (KEMRI) national ethical review committee (Reference No. KEMRI/RES/7/3/1). Participants were assured of confidentiality, informed consent was sought from, and participation was voluntary.

To assess whether one was HIV/AIDS affected, respondents were asked: *Have you personally been affected by HIV/AIDS *(Yes/No)? A follow up question: "*How have you been personally affected by HIV/AIDS?" *with multiple response options was then asked to those who answered "yes" to the first one. Responses included: i) currently caring or cared for someone infected with HIV/AIDS, ii) currently caring for orphaned children, iii) loss of support from adult children who died of, or currently sick with, AIDS, iv) self-reported HIV/AIDS infection, v) loss/reduction of community support to older people as a result of the HIV/AIDS epidemic, vi) loss of spouse to AIDS, vii) any other way. The above response categories were based on findings from a qualitative study in which older people in the same study area described the ways in which they had been affected by HIV/AIDS.

From the assessment of being HIV-affected, we derived binary exposure variables for the different ways in which older people could be affected by HIV/AIDS. To derive exposure status, respondents were first grouped into two broad categories of those affected and those not affected at all by HIV/AIDS. The former were then re-categorised according to the way in which they were affected. Respondents affected by HIV/AIDS in ways other than the one under investigation in individual models were not included in the specific analyses. For instance, for the exposure variable "HIV-infected" (x = 1), those who were affected by providing care to HIV/AIDS infected individuals or by providing care and support to orphans or those who had lost support from family and community were *not *included in the category of being unaffected (x = 0). This procedure was used in defining all the other exposure variables.

Health status was assessed using the short form of the individual SAGE (Study on Global AGEing and Adult Health) questionnaire [27]. This form has sections on health state descriptions in eight domains of health: mobility, self-care, affect, vision, pain and discomfort, sleep and energy, interpersonal activities and cognition.

In addition, the SAGE form has questions on functioning assessment using items in the Activities of Daily Living/Instrumental Activities of Daily Living (ADL/IADL) tool as well as on subjective wellbeing and quality of life.

For purposes of this analysis we used two measures of health status:

i) Self-rated health status which is derived from the question: *In general, how would you rate your health today, would you say your health is Very good (1), Good (2), Fair (3), Bad (4), or Very bad (5)*? A three-category outcome variable for Good (very good and good), Fair (fair) and Poor (very bad and bad) health was derived by collapsing the categories in parentheses.

ii) Composite health scores derived using item response theory (IRT) parameter estimates in Winsteps^®^, a Rasch measurement software package . IRT uses Maximum Likelihood Estimation methods to model the relationship between a person's health status and their probability of responding to each question in a multi-item scale. Typically, questions ask about how much difficulty the respondent had had in the preceding 30 days with tasks or activities in the eight domains. Responses range from no difficulty to extreme difficulty on a five-item scale. An example of a question in the mobility domain is: "*Overall in the last 30 days how much difficulty did you have with moving around?*" for which the response items were: 1(None), 2(Mild), 3(Moderate), 4(Severe), 5(Extreme/cannot do). Each item is modeled to have a set of parameters which describe the relationship between the item and the measured construct as well as how the item functions within a population. The health score is then transformed to a scale of 0 to 100 (where 100 is the best health status). More details on the application of the IRT approach to computing patient-reported health outcomes are available in the paper by Chang and Reeve [28]. Health scores were then compared between a) those affected by HIV/AIDS and those not and b) those affected by HIV/AIDS in different ways and those not affected in any way.

For the first measure of health, ordinal logistic regressions were carried out using HIV-affected status and the different ways of being affected as the exposure variables controlling for sex, age (age groups of 50–59, 60–69, 70–79, and 80+), marital status (in partnership, separated, divorced, never married and widowed), education level (no formal education, ≤ 6 years of school, >6 years of school), wealth index (quintiles), ethnicity (Kikuyu, Luo, Kamba, Luhya and others), and slum of residence.

For the second measure of health, linear regression was performed using the composite health score as the outcome variable. The regression model also included the covariates listed above in the same format. In both analyses, interaction terms between sex and the main explanatory variables were tested and none were found significant. All analyses were done in STATA^® ^Version 9.

## Results

There are many more males than females in the study population and the sex ratio is 1.00 to 0.56. More than two thirds of males are in the 50–59 age group compared to a little over half of females in the same age group. There are also more females in the 70+ age groups compared to males. Overall, 68.0% of respondents are married or have a partner though there are large sex differences with only 30.3% of females being in partnership compared to 89.1% of males. The majority of females are formerly married (widowed, divorced or separated) with widows accounting for more than two fifths of the entire sample of females. Almost half of the females have no formal education compared to less than 20% of males. Females are more likely to report being affected by HIV/AIDS than males. More details can be found in the descriptive characteristics table in additional file 1.

### HIV-Affected status

In all ways in which one can be HIV-affected, females were twice as likely to be affected as males except for perceived loss of community support. Overall <1% of respondents reported being HIV infected, 7.7% had cared for or were currently caring for a family member with AIDS, 5.3% had cared for or were currently caring for orphans, 1.8% had lost social and financial support due to the illness and/or death of their adult children, 4.0% had lost general community support normally given to older people and 0.3% had lost a spouse to HIV/AIDS. About 5% of respondents were affected in more than one way and in total 17.8% were affected by HIV/AIDS in at least one way.

### Self-reported health status

Overall, 59.7% of respondents reported having good health, 26.6% reported fair health and 13.7% reported poor health (males 8.9% and females 22.2%). There were noticeable differences between those affected by HIV and those not affected in these proportions. The respective percentages were 54.6, 28.9 and 16.5 for the HIV affected and 60.8, 26.1 and 13.1 for those not affected. The distribution of older people who reported bad or very bad health in different categories of key covariates is shown, in the descriptive characteristics table in additional file 1, separately for males and females. A higher proportion of females reported being in poor health than males and twice as many females in each age group reported being in poor health as males except for the 80+ age group. There was a steep age gradient with almost three times as many people in the 80+ age groups reporting poor health as those in the 50–59 age groups. There were noticeable sex differences in the proportions of respondents reporting poor health by marital status. An education gradient was also observed whereby a higher proportion of respondents with no formal education reported being in poor health than those with formal education for both males and females. For socioeconomic status, an inverted-U pattern was observed among females whereby the poorest and least poor were less likely to report being in poor health. The pattern was similar among males though with a different peak.

### Composite health scores

The overall mean health score was 70.6 (SD 13.9) and the median score was 67.5. For females the mean score (SD) and median score were respectively 66.1 (12.4) and 64.2, while for males they were 73.1 (14.1) and 69.5. HIV/AIDS affected respondents had slightly lower health scores (mean 68.5) than those unaffected (mean 71.1; t = 3.21, p = 0.0007). Respondents who were HIV infected had lower health scores than those not affected by HIV in any way (66.8 vs. 71.1) though the difference did not reach statistical significance. In the same way, those who had been affected through care giving for HIV/AIDS family members, care giving for orphans, loss of support through death of offspring, loss of community support, had significantly lower health scores than those who had not been affected in any way. The respective scores were 66.3 (t = 5.18, p = 0.000), 67.3 (t = 3.32, p = 0.0006), 66.0 (t = 2.70, p = 0.005) and 65.6 (t = 5.25, p = 0.000). The mean health scores for categories of key covariates are shown, in the table in additional file 1, separately for males and females. The pattern is similar to that of self-reported poor health for most covariates. Respondents in categories with higher proportions of people reporting poor health have lower health scores than the rest.

In unadjusted ordinal logistic regression models, HIV-affected respondents had higher odds of being in the poor health category than those not affected in any way with an overall odds ratio of 1.29 (95% CI: 1.04–1.61). Detailed results are shown in Table 1.

**Table 1:** Effect of different types of being HIV-affected on the self-reported health status of older people in the Nairobi DSS

**Exposure**^1^	**n**	**Crude OR**^2^ **(95% CI)**	**Adjusted OR**^2,3^ **(95% CI)**	**Results for other covariates^4^**
HIV Affected (Y/N)	370	1.29 (1.04–1.61)	1. 42 (1.12–1.80)	Sex*, age*, education level ^n.s.^, wealth index^n.s.^, marital status^n.s.^, slum of residence*, ethnicity ^n.s.^.
Self-reported HIV-Infected (Y/N)	17	1.65 (0.68–4.00)	1.45 (0.56–3.80)	Sex*, age*, education level ^n.s.^, wealth index^n.s.^, marital status^n.s.^, slum of residence*, ethnicity ^n.s.^.
Care for HIV/AIDS affected persons (Y/N)	160	1. 48 (1.09–2.01)	1.60 (1.15–2.23)	Sex*, age*, education level ^n.s.^, wealth index^n.s.^, marital status^n.s.^, slum of residence**, ethnicity ^n.s.^.
Care for HIV/AIDS orphans (Y/N)	109	1.30 (0.89–1.89)	1.24 (0.82–1.86)	Sex*, age*, education level ^n.s.^, wealth index^n.s.^, marital status^n.s.^, slum of residence***, ethnicity ^n.s.^
Lost support from deceased offspring (Y/N)	38	0.62 (0.3–1.29)	0.60 (0.28–1.29)	Sex**, age*, education level ^n.s.^, wealth index^n.s.^, marital status^n.s.^, slum of residence**, ethnicity ^n.s.^.
Lost community support (Y/N)	84	1.38 (0.89–2.12)	1.48 (0.94–2.36)	Sex**, age*, education level ^n.s.^, wealth index^n.s.^, marital status^n.s.^, slum of residence**, ethnicity ^n.s.^.

^1 ^Being HIV-affected through loss of spouse to AIDS not analyzed due to very small numbers

^2 ^Odds Ratio for being in the highest outcome category (poor health)

^3 ^Adjusted for sex, age group, marital status, education, wealth index, ethnicity and slum of residence

^4 ^* means that p < 0.001, ** means that p < 0.01, *** means that p < 0.05, n.s. means that p > 0.05

Respondents affected by HIV in all ways, except loss of support from deceased offspring and relatives, showed higher odds of being in the poor health category in unadjusted models. Only the odds ratio for those who were affected through care for HIV/AIDS affected family members reached statistical significance. In adjusted models, these results were only slightly changed. The odds ratios for being HIV/AIDS affected in any way and for care giving for ill family members were enhanced in adjusted models and retained statistical significance.

In all the adjusted ordinal logistic regression models, sex, age and slum of residence had significant effects on self-reported health. Females were more likely to report being in poor health than males, there was a positive age gradient whereby older respondents were more likely to report being in poor health than younger ones and residents of Korogocho were more likely to report being in poor health than those of Viwandani.

In unadjusted linear regression models, respondents who were HIV-affected in any way had lower mean health scores than those who were not, as shown by the negative β-coefficients (Table 2). Scores also tended to be lower among respondents affected by HIV in all the individual ways. The lowest relative health scores were observed among those affected through perceived loss of community support.

**Table 2:** Effect of different types of being HIV-affected on composite health scores of older people in the Nairobi DSS

**Exposure^5^**	**α**	**Crude β^6 ^(95% CI)**	**α**	**Adjusted^7 ^β(95% CI)**	**Results for other covariates^8^**
HIV Affected (Y/N)	**'**73.6	-2.53 (-4.09 – -0.97)	75.3	-2.94 (-4.46–-1.43)	Sex**, age*, education level ***., wealth index***^.^, marital status^n.s.^, slum of residence*, ethnicity ^n.s.^.
Self-reported HIV-Infected (Y/N)	71.1	-4.25 (-11.03 – 2.53)	73.1	-6.38 (-12.97 – 0.21)	Sex*, age*, education level ***., wealth index***^.^, marital status^n.s.^, slum of residence*, ethnicity ^n.s.^.
Care for HIV/AIDS affected persons (Y/N)	71.1	-4.75 (-7.01 – -2.50)	73.0	-4.82 (-6.99 – -2.65)	Sex**, age*, education level ***., wealth index***^.^, marital status^n.s.^, slum of residence*, ethnicity ^n.s.^.
Care for HIV/AIDS orphans (Y/N)	71.1	-3.73 (-6.45 – -1.02)	72.8	-2.97 (-5.61 – -0.34)	Sex*, age*, education level ***., wealth index***^.^, marital status^n.s.^, slum of residence*, ethnicity ^n.s.^.
Lost support from deceased offspring (Y/N)	71.1	-5.10 (-9.64 – -0.56)	72.5	-4.51 (-8.85 – -0.17)	Sex**, age*, education level ***., wealth index***^.^, marital status^n.s.^, slum of residence*, ethnicity ^n.s.^.
Lost community support (Y/N)	71.1	-5.47 (-8.53 – 2.41)	73.7	-5.88 (-8.83 – 2.92)	Sex**, age*, education level ***., wealth index***^.^, marital status^n.s.^, slum of residence**, ethnicity ^n.s.^.

^5 ^Being HIV-affected through loss of spouse to AIDS not analyzed due to very small numbers

^6 ^β = linear regression coefficients

^7 ^Adjusted for sex, age group, marital status, education, wealth index, ethnicity and slum of residence

^8 ^* means that p < 0.001, ** means that p < 0.01, *** means that p < 0.05, n.s. means that p > 0.05

In adjusted linear regression models, regression coefficients were mostly enhanced and hence health scores were lower than in unadjusted models. Significantly lower health scores were observed among those affected by HIV/AIDS in any way and those affected in all specific ways except those with self-reported HIV infection and perceived loss of community support.

In all the linear regression models, sex, age, slum of residence, education level, and wealth index had significant effects on the health score. The direction and magnitude of the effects were similar to those in the ordinal logistic regression models above for the first three covariates. There was an education gradient whereby those with more than six years of formal education had the highest health scores and those with no formal education the least. For wealth index, a non-monotonic relationship was observed whereby respondents in the first and fourth quintiles had the lowest health scores and those in the second had the highest.

## Discussion

This study aimed to assess the effect of HIV/AIDS on the health of older people in two Nairobi slums. More than one sixth of respondents aged 50 and over, were affected by HIV/AIDS in one way or the other mostly through care giving for HIV/AIDS-infected family members and HIV/AIDS orphaned children. About 14% of all respondents reported bad or very bad self-rated health with about 17% of the HIV-affected respondents reporting the same. There were significant differences in the mean health scores of those affected by HIV and those not affected, suggesting that HIV-affected older people have worse health outcomes than those not affected.

The sex and age distribution of the study population is atypical and is unlike what one would expect in the general population. It however reflects the nature of the two informal settlements which are predominantly occupied by labor migrants, most of whom are young males. In all age groups in the NUHDSS, after the age of 25 years, there are almost twice as many males as females migrating to or residing in the two informal settlements and this is also true for the 50+ age group. The high proportion of formerly married females reflects the underlying reasons for female migration into the slums which include marital strife. In addition, while most males remarry after the death of their spouse, few females do so in this setting, especially at older ages.

There are few studies that have assessed the health and wellbeing of older people in an African setting despite the increasing realization that population aging in the continent is a key demographic trend that will have significant impact on social support and fledgling health systems [1,20]. Few studies have assessed the impact of HIV/AIDS on the wellbeing of older people albeit in a qualitative manner [4,5]. This study therefore offers useful insights both on the overall health of older people in the study setting and the impact of being HIV/AIDS affected on the health of older people.

Generally, the absence of studies in similar settings makes it hard to interpret some of the findings. For instance, it is difficult to judge whether 13.7% – the proportion of respondents who reported having bad or very bad health – is high or low or is as expected. In addition, the absence of data on a comparison population and an assessment of contextual factors (if such a population existed) make it difficult to interpret the mean composite health score of 70.6 (we) observed in this study population. However, differences observed between older and younger respondents, between males and females as well as the expected gradient with educational status point to a high degree of internal validity. Gender differences in health status have been observed in studies in other settings where females fare worse than males [29-31] and so our findings of gender differences that persist in multivariate models are not surprising.

HIV-affected respondents exhibited lower mean health scores and had higher odds of being in the poor health category. However, the small number of respondents in the different exposure categories led to lack of significance in most estimates in the ordinal logistic regression models.

The finding that older people in care giving roles for HIV-infected family members have worse health outcomes than those not affected by HIV in any way is unsurprising. This could be explained by the relatively higher physical and emotional demands on care givers for terminally ill family members as has been found in other studies [4,18,20]. Care giving for orphans also has negative effects on health though the magnitude of the effect is slightly less than that from care giving for ill relatives and family members.

Some older people reported they had been affected by HIV/AIDS through loss of community support and neglect of the day-to-day needs of older people. Findings from other studies in this community suggest that community support is largely limited to major needs such as funerals and hospitalization but not for what may often be considered routine needs such as food or minor illnesses [32]. While the fragmented nature of social relationships in urban informal settlements may lead to an overall reduction in community support, it is possible that the HIV/AIDS epidemic may have worsened the situation. The importance of this result, the low percentages notwithstanding, is that it highlights another way in which the community perceives being HIV/AIDS affected other than the more established ways such as care giving roles and loss of support from ill or deceased family members. The results highlight the need for services and programs that target all vulnerable groups in the community rather than those favored by most programs.

The proportion of respondents that were or had been involved in care giving roles as well as the proportion of older people reporting being affected by HIV through loss of support from deceased or ill family members are surprisingly low given the high burden of HIV/AIDS in the community [25]. However, this may be explained by the fact that a high proportion (23.9%) of older people lives alone or with just their spouses and they do not have extensive social or familial networks as would be expected in a rural area or non-slum urban area. It is possible that few adult children live with or support their parents in this setting and hence, the high AIDS mortality notwithstanding, few older people are affected in this way.

The self-reported HIV prevalence of 0.8% in this population is very low and is most probably an underestimate given the fact that the HIV prevalence in the adult population in the two slums (11.5%) [24] is much higher than the national average (7.4%) [12] and that HIV-related mortality in this population is very high [25]. In addition, recent estimates of HIV prevalence in the country show a level of about 8% for the 50–54 age group, 4% for the 55–59 age group, and about 2.5% for the 60–64 age group [12]. The majority of our study participants fall in the age range of 50–69 years and hence the true HIV prevalence could be higher than was reported. This small proportion of self-reported HIV infection could also point to the low uptake of HIV counseling and testing services among this age group and/or the lack of HIV/AIDS related programs targeting older people or simply unwillingness on respondents' part to voluntarily disclose their HIV sero-status. Unknown or unreported HIV status therefore potentially biases our estimates if some true HIV positive respondents are classified as not being affected by HIV in any way. This has the effect of underestimating poor health status among the "unexposed" and hence underestimating the true relative effect of being HIV-affected. In reality, therefore, the impact of being HIV-affected on health could even be worse than estimated by our study.

Self-reported health status, though widely used in other settings, has not been as widely used in African settings and its validity as a measure of health has not been established. It is known that the validity of self-reported measures of health and their reliability are influenced by underlying socio-cultural factors including basic and health literacy, cultural perceptions of illness, disability and health among others [33,34]. The finding of steep age and education gradients and worse female health scores, however, point to a good degree of internal validity. Further studies including vignettes should investigate the influence of contextual factors on the validity of self-reported health in this population.

The use of a composite measure of health masks difficulties with activities in individual domains of health or domain-specific health outcomes. For instance, the study did not investigate the extent to which variations in mean health scores could be dependent on particularly large scores in domains such as pain and discomfort, affect and sleep which are likely to be more affected if one is HIV-affected compared to domains like cognition, vision and breathing. Given the large number of domains involved (eight), it would have been impossible to do justice to all of them in one paper and show how, difficulty in each domain, could be explained by the various ways of being HIV-affected. The sample size is also too small for such detailed analyses.

This being a cross-sectional study, temporality cannot be established. It is possible that there is a selection effect, for example, among respondents who cared for HIV/AIDS infected relatives or those who cared for orphans. Older people in poor health would not be expected to care for relatives or orphans unless no other relative was available. Longitudinal studies that establish health state dynamics as a function of being HIV-affected taking selection effects into account are recommended.

## Conclusion

The importance of older people in the fight against HIV/AIDS cannot be overemphasized. Our study, the first of its kind in the African region aimed to demonstrate the effect of being HIV-affected on the health of older people. The worse health outcomes exhibited by older people affected by HIV/AIDS highlight the need for policy makers, development partners and civil society organizations involved in HIV/AIDS initiatives to develop policies and programs that specifically target older people. Such programs should include those that improve HIV/AIDS and other general health services for older people and those that increase uptake for counseling and testing services. Older people should also be targeted by initiatives aimed at mitigating the social and economic impact of HIV/AIDS on vulnerable groups.

## Competing interests

The authors declare that they have no competing interests.

## Authors' contributions

CK contributed to the design of the study instrument, did the data analysis and literature review, wrote the first draft and contributed to the interpretation of the results, ACE contributed to the conceptualization of the study, writing the paper and interpretation of the findings, EZ conceptualized the study, contributed to the writing of the paper and interpretation of the findings, JF contributed to the overall conceptualization of the study and the design of the survey instrument, contributed to writing the paper and interpretation of the findings. All authors read and approved the final manuscript.

## Pre-publication history

The pre-publication history for this paper can be accessed here:



## Supplementary Material

Additional file 1 **Descriptive characteristics table**. Descriptive characteristics of the study population of older people in the Nairobi DSS, November 2006 to February 2007 Click here for file
