# Supplementary material for: HIV/AIDS and the health of older people in the slums of Nairobi, Kenya: results from a cross sectional survey
Source: BMC Public Health. 2009 May 27;9:153. doi: 10.1186/1471-2458-9-153 (PMC2694783; doi:10.1186/1471-2458-9-153)
Supplement: Additional file 1 — Descriptive characteristics table. Descriptive characteristics of the study population of older people in the Nairobi DSS, November 2006 to February 2007 [file 1471-2458-9-153-S1.doc]

## Descriptive characteristics of the study population of older people in the Nairobi DSS, November 2006 to February 2007

|  |  | **Males** | | | | **Females** | | | |
| --- | --- | --- | --- | --- | --- | --- | --- | --- | --- |
| **Variables** | **Categories** | **n** | **%** | **Mean health score** | **% with “poor” self-reported health** | **n** | **%** | **Mean health score** | **% with “poor” self-reported health** |
| Age group | 50-59 | 944 | 70.9 | 74.7 | 7.0 | 418 | 56.0 | 69.6 | 14.1 |
|  | 60-69 | 270 | 20.3 | 71.0 | 9.6 | 189 | 25.3 | 63.9 | 23.8 |
|  | 70-79 | 79 | 5.9 | 69.0 | 17.7 | 85 | 11.4 | 60.0 | 45.9 |
|  | 80+ | 38 | 2.9 | 59.3 | 34.2 | 55 | 7.4 | 56.6 | 41.8 |
| Marital status | Married/has partner | 1186 | 89.1 | 73.7 | 7.4 | 226 | 30.3 | 68.8 | 12.8 |
|  | Separated | 41 | 3.1 | 66.0 | 24.4 | 92 | 12.3 | 67.9 | 17.4 |
|  | Divorced | 19 | 1.4 | 68.9 | 15.8 | 50 | 6.7 | 66.1 | 30.0 |
|  | Widowed | 66 | 5.0 | 68.2 | 25.8 | 315 | 42.2 | 63.2 | 29.2 |
|  | Never married | 19 | 1.4 | 71.9 | 5.3 | 64 | 8.6 | 66.2 | 21.9 |
| Wealth Index | 1^st^ Quintile (Poorest) | 383 | 28.8 | 72.7 | 7.1 | 125 | 16.7 | 67.4 | 21.6 |
|  | 2^nd^ Quintile | 153 | 11.5 | 73.4 | 17.7 | 170 | 22.8 | 64.6 | 24.7 |
|  | 3^rd^ Quintile | 250 | 18.8 | 74.5 | 8.4 | 169 | 22.6 | 65.0 | 30.8 |
|  | 4^th^ Quintile | 254 | 19.0 | 71.2 | 9.8 | 180 | 24.1 | 66.5 | 16.1 |
|  | 5^th^ Quintile | 290 | 21.8 | 74.1 | 6.6 | 103 | 13.8 | 68.4 | 15.5 |
| Education Status | No formal education | 235 | 18.1 | 69.2 | 15.7 | 337 | 48.5 | 63.9 | 27.0 |
|  | ≤ 6 years | 363 | 27.9 | 71.9 | 12.7 | 199 | 28.6 | 67.9 | 19.6 |
|  | > 6 years | 704 | 54.1 | 75.0 | 4.8 | 159 | 22.9 | 70.5 | 10.1 |
| HIV/AIDS affected? | No | 1125 | 84.6 | 73.5 | 8.8 | 582 | 77.9 | 66.5 | 21.5 |
|  | Yes | 205 | 15.4 | 71.4 | 9.8 | 165 | 22.1 | 65.0 | 24.9 |
|  | Yes – self-reported HIV infection | 8 | 0.6 | 75.1 | NA | 9 | 1.2 | 59.5 | NA |
|  | Yes – care giving for ill family members | 78 | 5.9 | 68.4 | NA | 82 | 11.0 | 64.3 | NA |
|  | Yes – care giving for orphans | 50 | 3.8 | 71.1 | NA | 59 | 7.9 | 64.2 | NA |
|  | Yes – loss of family support | 14 | 1.1 | 71.9 | NA | 24 | 3.2 | 62.5 | NA |
|  | Yes – loss of community support | 55 | 4.1 | 66.2 | NA | 29 | 3.9 | 64.4 | NA |
|  | Yes – loss of spouse to AIDS | 3 | 0.2 | 73.9 | NA | 3 | 0.4 | 64.9 | NA |
| **All** |  | **1331** | **64.1** | **73.1** | **8.9** | **747** | **35.9** | **66.1** | **22.2** |
